# A newborn tolerated severe hypercapnia during general anesthesia: a case report

**DOI:** 10.1186/s13256-015-0685-6

**Published:** 2015-09-14

**Authors:** Kai Wei, Hui Xu, Wanmin Liao, Chuanhan Zhang, Wenlong Yao

**Affiliations:** Department of Anesthesiology, Tongji Hospital, Tongji Medical College, Huazhong University of Science and Technology, 1095 Jiefang Avenue, Wuhan, 430030 China

## Abstract

**Introduction:**

Severe hypercapnia is a rare but harmful complication of general anesthesia. We report the case of a newborn who developed severe hypercapnia with unknown reasons during general anesthesia but recovered well. This report will advance our understanding about the causes of severe hypercapnia during anesthesia, the possible compensatory mechanisms and the characteristics of neonatal respiratory physiology and intracellular buffering systems.

**Case presentation:**

A 21-day-old Chinese baby girl who had an incarcerated hernia received an emergent exploratory operation under general anesthesia. She developed severe hypercapnia during surgery for unclear reasons. Arterial blood gas revealed a PCO_2_ of 149mmHg. Troubleshooting and relevant measures were taken, but the level of CO_2_ did not decrease. In spite of the high level of PCO_2_, the newborn recovered well without any complications.

**Conclusions:**

Neonates are vulnerable to hypercapnia during anesthesia for their characteristic respiratory physiology. Heat and moisture exchange should be used with caution in newborns under general anesthesia as it can increase dead space. Intracellular buffering systems play an important role in tolerating severe hypercapnia. Although this case raised a great challenge to the homeostatic mechanism of the body, measures should be taken to maintain PCO_2_ values around the clinically acceptable level.

## Introduction

The process of lung maturation is not completed in a full-term newborn. A European cohort of ventilated newborn babies indicated that hypercapnia was more common than hypocapnia [1]. However, severe hypercapnia can also bring some adverse effects in neonates such as periventricular white matter injury, intraventricular hemorrhage and retinopathy of prematurity [2]. Here we present and analyze the case of a newborn who experienced severe hypercapnia during general anesthesia but recovered well with no complications.

## Case presentation

A 21-day-old Chinese baby girl weighing 3.8kg with an incarcerated hernia underwent emergent inguinal herniorrhaphy. The newborn was a full-term delivery and was born on mixed feeding without any special medical history. Anesthesia was induced uneventfully with propofol 2.5mg·kg^−1^ and fentanyl 2μg·kg^−1^. She was paralyzed with atracurium 0.5mg·kg^−1^ and intubated with a size 3.0 cuffed tracheal tube. She was ventilated with a time-cycled, pressure-limited ventilation mode using a pediatric circle circuit, with a respiratory rate of 25min^−1^ and maximum pressure (P_max_) of 15cm H_2_O_._ The machine showed the tidal volume given to the newborn was 45mL. Partial pressure of end-tidal carbon dioxide (P_ET_CO2) fluctuated between 35 and 45mmHg. Noninvasive blood pressure, electrocardiogram, pulse oximetry, P_ET_CO_2_ and body temperature were monitored continuously and these remained within normal levels throughout induction of anesthesia. Anesthesia was maintained with sevoflurane and remifentanil.

Twenty minutes after anesthesia, P_ET_CO_2_ increased from 45mmHg to 50mmHg and rose to 90mmHg in 30 minutes. The body temperature of the newborn was 37.5°C at the beginning of surgery. When P_ET_CO_2_ began to rise, she flushed and her body temperature increased to 38.3°C. The warm blanket was removed and the heating in the operating room was turned off. Ten minutes later, her body temperature was down to normal level.

Troubleshooting and relevant measures were taken when P_ET_CO_2_ began to rise. The depth of the endotracheal tube was checked by auscultating both lung fields. The CO_2_ absorber was checked and found to be of normal condition but was still replaced. Respiratory rate and P_max_ were adjusted to 40 min^−1^ and 18cm H_2_O respectively. Tidal volume showed 60ml at this time. Meanwhile, the CO_2_-analyzing line and CO_2_-analyzing water trap cassette were changed, but without any obvious change in P_ET_CO_2_. Neuromuscular blockers were added to exclude the recovery of spontaneous breathing and respiratory antagonism. Manual ventilation was applied to exclude any malfunction of the anesthesia machine. In spite of all these measures, P_ET_CO_2_ did not decrease.

Surprisingly, her heart rate and blood pressure fluctuated ± 20% of baseline during the increase of P_ET_CO_2_. The operation was not interrupted and finished rapidly as there was no intestinal ischemia. Femoral artery blood gas analysis at the end of the surgery showed severe respiratory acidosis (Table 1). Twenty minutes after the blood gas analysis, there was no obvious change in P_ET_CO_2_ but the newborn suddenly opened her eyes. She recovered spontaneous breathing. The anesthetic circuit was disconnected from the ventilator for 5 minutes and pulse oximetry saturation remained normal. So, the endotracheal tube was removed. After extubation, femoral vein blood gas analysis showed partial pressure of carbon dioxide (PCO_2_) of 115mmHg and the neonate was sent to the pediatric intensive care unit (PICU). Two hours later, a repeat artery blood gas analysis indicated that PCO_2_ was 31.8mmHg and pH was 7.352. Postoperative review showed there were no complications.

**Table 1:** Artery blood gas at the end of the surgery

pH	pO_2_	pCO_2_	BE	Hb	Hct	K^+^	Na^+^	Glu
6.831	94.6mmHg	149mmHg	−9.8mmol/L	11.4g/dL	35%	5.4mmol/L	127mmol/L	15.1mmol/L

## Discussion

In this newborn, the main problem noted was severe hypercapnia. Infants are more likely to develop hypercapnia because of the age-related physiological changes in vital systems and a higher metabolic rate in relation to weight. The causes of hypercapnia include two aspects: the increase of CO_2_ generation and the decrease of CO_2_ discharge. Detailed factors include malignant hyperthermia, equipment and breathing circuit malfunction, patients’ preexisting pulmonary diseases, CO_2_ pneumoperitoneum, improper ventilatory settings and invalidity of CO_2_ absorber. However, in the present case, we failed to determine the real cause of the hypercapnia. Malignant hyperthermia was excluded as the neonate’s temperature decreased to normal level after the heating was switched off in the operating room and she did not show any sign of muscle rigidity, tachycardia, etc. CO_2_ canisters as well as the anesthesia machine were working properly. Time-cycled, pressure-limited ventilation mode is the most widely used mechanical ventilation mode for neonates and ventilation parameters were appropriate for the newborn.

An important factor we ignored during anesthesia was that a heat and moisture exchanger (HEM) was used between the circle system Y-piece and endotracheal tube. A circuit containing HEM can increase the dead space by almost 70% compared with a circuit without HEM in a 24-month-old child undergoing general anesthesia [3]. We did not realize the HEM was there until the tracheal extubation. It is not certain whether the HEM caused hypercapnia in this neonate, but a HEM used in pediatric anesthesia can induce hypoventilation with subsequent hypercapnia [3].

Hypercapnia is a two-edged sword and it exerts multiple physiologic effects on different organs. The word “permissive hypercapnia” was first described by Hickling in 1990 who suggested that adoption of low-tidal-volume ventilation strategies with tolerance of elevation in PCO_2_ was associated with lower hospital mortality [4]. Hypercapnia displaces the oxygen dissociation curve to the right, which improves tissue oxygenation and perfusion and may alleviate the injury to immature lung and brain [5]. Retrospective studies in premature neonates also concluded that higher PCO_2_ contributed to less lung injury [6].

In spite of these protective effects, it is believed that severe hypercapnia leads to an increase of intracranial pressure (ICP) via acidosis-induced cerebral arteriolar dilation, which is thought to be related with well-known neurological complications such as cerebral edema, intraventricular hemorrhage and temporary neurological impairment [7]. Reduced skeletal muscle contractility and decreased myocardial contractility are also known to occur following hypercapnia [8]. However, no evidence of neurological sequelae was observed in this neonate.

A few cases of severe hypercapnia have been reported in the literature. Most cases of patients with severe hypercapnia are related to chronic obstructive pulmonary disease, exacerbation of bronchial asthma causing severe hypoxemia or defective apparatus used during general anesthesia [9, 10]. Compared with those patients, several unique characteristics in our case deserve further discussion. First, to the best of our knowledge, this is the first reported case of a neonate who developed severe hypercapnia during general anesthesia for unclear reasons. Second, the neonate regained full consciousness and no residual neurological deficit was observed.

The ability to tolerate hypercapnia varies among individuals for a broad spectrum of factors such as duration of hypercapnia, the speed at which high values are reached, maximal PCO_2_ value, patient age and preexisting diseases. The threshold of PCO_2_ above which neurological impairment occurs is unclear [11]. Hypercapnia coma has been observed in patients with PCO_2_ values of 58mmHg. However, a sober patient with PCO_2_ values of 160mmHg has also been reported [12].

Remarkable renal compensation enables patients with end-stage lung disease to tolerate severe chronic hypercapnia. But how to explain why the newborn can tolerate such severe hypercapnia? The first reason could be that the neonate is insensitive to elevation of CO_2._ Central chemosensitivity in the newborn is not the same as that in the adult. In term neonates, the ventilatory handling of a hypercapnia challenge is fully functional on postnatal day 2. Then during the following 8 weeks, ventilatory response to CO_2_ declines markedly and subsequently achieves adult level of responsiveness after the 9th week [13]. The altered CO_2_ sensitivity of neurons, the loss of GABAergic excitation and changes in astrocytic function during development may account for the triphasic pattern of responsiveness to the CO_2_ [14]. Both carotid and central chemoreceptors are important for a normal ventilatory of CO_2_–H^+^ chemosensitivity. Nevertheless, carotid plays a greater role at low levels of hypercapnia, whereas intracranial chemoreceptors are more dominant at high levels [15]. In neonates, peripheral CO_2_ chemoreceptors exert greater influence than central CO_2_ chemoreceptors [13]. Therefore, we may infer that a nadir of ventilatory responsiveness to CO_2_ in this 21-day-old newborn as well as the specificity of CO_2_ chemoreceptors in neonates might make newborns insensitive to elevation of CO_2_. This might be an important reason why the neonate can tolerate severe hypercapnia.

Clinical severe hypercapnia frequently occurred with tissue hypoxia or arterial hypoxemia. However, in this newborn, this is not the case. Oxygen saturation was continuously monitored and it never fell below 95% during the increase of P_ET_CO_2_. Hemodynamics remained stable during the anesthetic procedure, which also guaranteed tissue perfusion and oxygenation. Research has indicated that the central system has a potent ability to recover from nonhypoxic severe respiratory acidosis [9]. As long as tissue perfusion and oxygenation are preserved, effective transfer of HCO_3_^−^ from extracellular space into cells by Na^+^/K^+^ changes or Na^+^/H^+^ changes will continuously occur when exposing to hypercapnia [10]. Consequently, the drop of pH in intracellular cells is far slower than that in extracellular cells. It is important to note that this regulation is energy-consumed, which requires the cells to be oxygenation and perfused [10]. The adequate oxygenation and strong chemical buffering capacity in intracellular cells enable important organs to tolerate extreme acidosis and that might contribute to the benign outcome of the newborn.

## Conclusions

Gross hypercapnia occurs rarely during general anesthesia. Heat and moisture exchange should be used with caution in newborns under general anesthesia as it can increase dead space. This study will significantly advance our understanding about the characteristics of neonatal respiratory physiology, intracellular buffering systems, and causes of severe hypercapnia. Although this case raised a great challenge to the homeostatic mechanism of the body, measures should be taken to maintain PCO_2_ values around the clinically acceptable level.

## Consent

Written informed consent was obtained from the patient’s legal guardians for publication of this case report and any accompanying images. A copy of the written consent is available for review by the Editor-in-Chief of this journal.
